# The Effect of SERCA1b Silencing on the Differentiation and Calcium Homeostasis of C2C12 Skeletal Muscle Cells

**DOI:** 10.1371/journal.pone.0123583

**Published:** 2015-04-20

**Authors:** Adrienn Tóth, János Fodor, János Vincze, Tamás Oláh, Tamás Juhász, Róza Zákány, László Csernoch, Ernő Zádor

**Affiliations:** 1 Department of Physiology, Faculty of Medicine, University of Debrecen, Debrecen, Hungary; 2 Department of Anatomy, Histology and Embryology, Faculty of Medicine, University of Debrecen, Debrecen, Hungary; 3 Department of Biochemistry, Faculty of Medicine, University of Szeged, Szeged, Hungary; Cinvestav-IPN, MEXICO

## Abstract

The sarcoplasmic/endoplasmic reticulum Ca^2+^ATPases (SERCAs) are the main Ca^2+^ pumps which decrease the intracellular Ca^2+ ^level by reaccumulating Ca^2+^ into the sarcoplasmic reticulum. The neonatal SERCA1b is the major Ca^2+^ pump in myotubes and young muscle fibers. To understand its role during skeletal muscle differentiation its synthesis has been interfered with specific shRNA sequence. Stably transfected clones showing significantly decreased SERCA1b expression (cloneC1) were selected for experiments. The expression of the regulatory proteins of skeletal muscle differentiation was examined either by Western-blot at the protein level for MyoD, STIM1, calsequestrin (CSQ), and calcineurin (CaN) or by RT-PCR for myostatin and MCIP1.4. Quantitative analysis revealed significant alterations in CSQ, STIM1, and CaN expression in cloneC1 as compared to control cells. To examine the functional consequences of the decreased expression of SERCA1b, repeated Ca^2+^-transients were evoked by applications of 120 mM KCl. The significantly higher [Ca^2+^]i measured at the 20^th^ and 40^th^ seconds after the beginning of KCl application (112±3 and 110±3 nM *vs*. 150±7 and 135±5 nM, in control and in cloneC1 cells, respectively) indicated a decreased Ca^2+^-uptake capability which was quantified by extracting the maximal pump rate (454±41 μM/s *vs*. 144±24 μM/s, in control and in cloneC1 cells). Furthermore, the rate of calcium release from the SR (610±60 *vs*. 377±64 μM/s) and the amount of calcium released (843±75 μM *vs*. 576±80 μM) were also significantly suppressed. These changes were also accompanied by a reduced activity of CaN in cells with decreased SERCA1b. In parallel, cloneC1 cells showed inhibited cell proliferation and decreased myotube nuclear numbers. Moreover, while cyclosporineA treatment suppressed the proliferation of parental cultures it had no effect on cloneC1 cells. SERCA1b is thus considered to play an essential role in the regulation of [Ca^2+^]i and its *ab ovo* gene silencing results in decreased skeletal muscle differentiation.

## Introduction

Skeletal muscle development is based on the fusion of myoblasts into a myotube. This multinucleated syntitium contains a complex and sophisticated internal membrane system called sarcoplasmic reticulum (SR) considered as a specialized form of endoplasmic reticulum (ER; reviewed by [1]). The SR is an attribute of muscle entity and predominantly regulates calcium movements during contraction-relaxation cycle; Ca^2+^ is released from the SR into the sarcoplasmic space where it triggers muscle contraction then it is reuptaken during the relaxation period and stored in the SR. There are proteins in the SR specialized for this activity; the main players being the ryanodine receptor (RyR) through which Ca^2+^ is released into the sarcoplasm, the sarcoplasmic/endoplasmic reticulum Ca^2+^-ATPase (SERCA) that reuptakes Ca^2+^ into the SR from the sarcoplasm, and calsequestrin (CSQ) that binds stored Ca^2+^ in the SR lumen. The three main SR proteins are expressed in developmental isoforms in fetal/postnatal stages and in myotubes of mammals. RyR expressed as RyR3 [2], CSQ as CSQ2/cCSQ [3,4], and SERCA as SERCA1b [3,4]. The ratio and the functional differences of these proteins compared to the adult isoforms are not entirely known although it could probably be important for better understanding the mechanism of muscle differentiation and store-operated calcium entry (SOCE).

SOCE, the process through which the SR is refilled with Ca^2+^ from the extracellular source once its content has been reduced, has been shown to be important in muscle development [5,6]. This underlying process of muscle differentiation is initiated by one of the stromal interaction molecule isoforms, STIM1 serving with its intraluminal part as a calcium sensor within the ER/SR [7]. In case of low Ca^2+^-level the luminal part of STIM1 monomers do not bind to Ca^2+^ in the ER/SR rather they associate with each other and are transferred to the close proximity of the plasma membrane where they activate Orai1, a channel allowing extracellular Ca^2+^-entry into the cell. Subsequently Ca^2+^ is transferred from the sarcoplasm to the SR by SERCA pump activity (reviewed by [8]).

The aim of present study was to explore the function of SERCA1b, a major calcium pump of *in vitro* myotubes and embryonic/postnatal human and rodent muscles [4,9]. SERCA1b mRNA is spliced from the transcript of the SERCA1 gene (atp2a1) by skipping exon 22 while in the adult SERCA1a mRNA each exon remains [10]. Since the first stop codon is in exon 22, the translation of SERCA1b terminates in exon 23 using the second stop codon. As a result, the SERCA1b protein has an eight amino acid long tail instead of the C-terminal glycine of the SERCA1a protein [3]. SERCA1a is expressed in adult fast type skeletal muscle, however, no functional difference could be observed in the Ca^2+^ transport and affinity if compared to SERCA1b when their corresponding cDNAs are expressed in COS-1 cells [11]. SERCA1 knock-out mice (expressing neither SERCA1a nor SERCA1b) die in respiratory failure and cyanosis shortly after birth probably because of insufficient function and development of the diaphragm [12], which has been shown to express SERCA1b as the main SERCA1 isoform in neonatal mice [4]. Interestingly, the expression of SERCA1b is under strict posttranscriptional control; although its mRNA is upregulated in stretch [13] and denervation [14] of adult muscle, the protein is expressed only in developing or regenerating muscle independently of whether it is becoming a slow or fast type [4].

The function of SERCA1b has partially been uncovered by injecting regenerating *m*. *soleus* of the rat with a plasmid expressing an shRNA targeted against its mRNA [15]. Although less than 1% of the fibers and even less than 0.1% of the myonuclei were successfully transfected [16] it remarkably stimulated growth also in the non-transfected fibers and in the entire regenerating muscle. Apparently this effect was initiated from the transfected fibers through autocrine-paracrine mechanisms probably through the calcineurin-NFAT-interleukin4 pathway because it was independently prevented by co-transfection with a calcineurin(CaN) inhibitor cain/cabin expressing vector and the perimuscular injection of interleukin4 (IL4) antibody. However, the decline of SERCA1b in the above study could only be demonstrated by immunohistochemistry in about 25% of the shRNA transfected fibers. It has also been show recently that the SERCA1a is bound and activity-stimulated by the stromal interactive molecule 1 (STIM1) in primary mouse myotubes [17]. As SERCA1b has the entire SERCA1a sequence except the C-terminal glycine [3] and is the dominant SERCA1 in mouse myotubes this also underline the importance of this neonatal Ca^2+^ pump.

Our hypothesis was that SERCA1b is an essential contributor to myotube development and other myogenic processes. Experiments were, therefore, conducted to explore the cellular effects of stable SERCA1b silencing in the myogenic C2C12 cells on the expression of proteins involved in calcium handling, on calcium homeostasis, and on muscle differentiation. SERCA1b was successfully silenced in 75% of stably transfected clones. The results presented here are complementary to the SERCA1b silencing in regenerating muscle [15] and show for the first time the importance of the neonatal calcium pump in calcium handling, proliferation, and myotube development of myogenic cells.

## Materials and Methods

### Cell cultures and transfection

C2C12 mouse skeletal muscle cell line was obtained from the European Collection of Cell Cultures (ECACC). The cells were cultured in Dulbecco’s Modified Eagle’s Medium (DMEM; Sigma-Aldrich, St. Louis, MO, USA) supplemented with 10% fetal bovine serum(FBS), 50 U/ml penicillin, and 50 g/ml streptomycin and were incubated at 37°C in an atmosphere of 95% air and 5% CO_2_ and 80% humidity in a humidified incubator (according to the instructions of the supplier). RNA interference was applied in order to reduce endogenous SERCA1b expression. The sequence targeted for SERCA1b silencing was 5-*ctatctggaggatccagaa*-3 (S1A Fig), corresponding to the last 11 bases of exon 21 (3121–3131b of the mouse SERCA1 cDNA, acc. No. NM_007504) and the first 8 bases of exon 23 (3174–81b). These fragments are contiguous only in the spliced SERCA1b mRNA after skipping exon 22. The chosen shRNA cassette sequence besides the sense (5’*ctatctggaggatccagaa*) and antisense (5’*ttctggatcctccagatag*) region contains a loop and termination sequence resulting in a hairpin siRNA. Blast filtering ensured that this sequence has homology only with SERCA1b and not with any other known gene. Scrambled shRNA was used to demonstrate that the target specific shRNA did not induce a nonspecific effect on gene expression. These shRNA sequences were cloned into pLKO.1-puro-CMV-tGFP expression vector. Stable transfection was performed in Opti-MEM reduced serum content medium using Lipofectamine 2000 reagent (Invitrogen, Carlsbad, CA, USA) for 2.5 h at 37°C. Cells were allowed to express the introduced sequence for 48 h in growth medium then were selected in DMEM containing 1.5 μg/ml puromycin. After 14–15 days, single colonies were isolated and experiments were carried out on separated clones of SERCA1b transfected cells. Pool of scrambled shRNA transfected and non-transfected parental cells were used as control. Differentiation of non-transfected, and transfected cells was induced at 80% confluency by exchanging the culture medium for DMEM supplemented with 5% horse serum (HS), 2% FBS and penicillin–streptomycin. The efficiency of SERCA1b gene silencing was monitored at protein level by Western-blot using a specific anti-SERCA1b antibody corresponding to the terminal octamer of the protein. Functional experiments were carried out on 5-day-old differentiated myotubes of parental and transfected cultures.

### Immunostaining

Cultured cells were washed with ice-cold phosphate-buffered saline (PBS; 0.02 M NaH_2_PO_4_, 0.1 M NaCl), fixed with −20°C 100% methanol for 15 min, permeabilized with 0.1% Triton X-100 in PBS for 10 min, and blocked with 1% bovine serum albumin (BSA) diluted in PBS (blocking solution) for 30 min at room temperature. Cells were then incubated for 4 hours at 4°C with the anti-desmin primary antibody (dilution was 1:500 in blocking solution). Then fluorescein (FITC) labeled anti-mouse secondary antibody was applied for 1 h at room temperature. Vectashield mounting medium with DAPI (Vector Laboratories, Burlingame, CA, USA) was used for visualization of nuclei. Images were taken using LSM 510 META confocal microscope (Zeiss, Oberkochen, Germany).

### Preparation of cell extracts

On the 5^th^ day of differentiation cells in cultures were washed with ice-cold PBS, harvested in homogenization buffer (20 mM Tris–Cl, 5 mM EGTA, 1 mM 4-(2-aminoethyl) benzenesulphonyl fluoride, 20 μM leupeptin, pH 7.4; all from Sigma) and disrupted by sonication on ice. Protein content of the samples was measured by a modified bicinchoninic acid (BCA) protein assay (Pierce, Rockford, IL, USA) using BSA as a standard.

For SERCA2a detection, on the 5^th^ day of differentiation cells in cultures were washed with ice-cold PBS, harvested in PBS and collected by centrifugation. The cells were washed twice with cold PBS. The supernatant were removed and discard, and collected the cell pellet. The cell pellet were lysed in cell lysis buffer (50 mM Tris–Cl, 5 mM EDTA, 250 mM NaCl, 50 mM NaF, 1 mM sodium orthovanadate, 1% Nonidet P40, 0.02% sodium azide, pH 7.4; all from Sigma) for 30 minutes, on ice, with vortexing at 10 minute intervals. The cell lysis buffer was supplemented with 1 mM phenylmethylsulfonyl fluoride (PMSF; 0.3 M stock in DMSO) and protease inhibitor cocktail. The extract were transferred to microcentrifuge tubes and centrifuged at 13,000 rpm for 10 minutes at 4°C. The clear lysate was measured by a modified bicinchoninic acid (BCA) protein assay (Pierce, Rockford, IL, USA) using BSA as a standard. Heat denaturation was applied at 80° C for 5 min

### SDS–PAGE and Western blot analysis

Total cell lysates were examined by Western-blot analysis. Samples for SDS–PAGE were prepared by the addition of 1/5 volume of 5-fold concentrated electrophoresis sample buffer (310 mM Tris–HCl, pH 6.8; 10% SDS, 50% glycerol, 100 mM DTT, 0.01% bromophenol blue) to cell lysates and boiled for 5 min at 80°C. 30 μg of protein was separated by 7.5% SDS–PAGE gel for immunological detection of examined proteins. Samples were transferred electrophoretically to nitrocellulose membranes (Bio-Rad Laboratories, CA, USA). After blocking with 5% non-fat dry milk in PBS, membranes were incubated with the appropriate primary antibodies overnight at 4°C (see Table 1). After washing three times for 10 min with PBST (PBS supplemented with 0.1% Tween 20), membranes were incubated with a secondary antibody, peroxidase-conjugated goat anti-rabbit, and anti-mouse IgG (BioRad) in 1:1000 dilution in PBS containing 5% non-fat dry milk for 1 h. Signals were detected by enhanced chemiluminescence (ECL) reaction (Thermo Scientific, Rockford, IL USA). The quantitative analysis was performed using the original images of the membranes. The intensity of the specific bands and the background from the same image were measured by ImageJ. The background values were then substracted. The values were normalized to those obtained for actins of the same samples. These data were then expressed as relative to control or scrambled. Measurements were carried out in 3 independent experiments.

**Table 1:** List of antibodies using for the detection of different proteins playing important role of calcium homeostasis and differentiation of skeletal muscle

name	commercial supplier	catalog number	host	source	dilutions	epitope
**MyoD**	Santa Cruz	sc-377460	mouse	monoclonal	1/200	amino acids 1–318 of MyoD of mouse origin
**SERCA1b**	Zádor E. Univ. of Szeged		rabbit	monoclonal	1/800	terminal octamer of rat SERCA1b
**SERCA1**	Thermo Scientific	MA3-912	mouse	monoclonal	1/1000	amino acid 506-C-terminus of rabbit skeletal muscle ATPase
**SERCA2a**	F. Wuytack Univ. of Rotterdam		rabbit	monoclonal	1/20000	amino acid 989–997 of pig SERCA2a isoform
**Calcineurin**	Cell Signaling	2614S	rabbit	polyclonal	1/1000	carboxy terminus of human calcineurin (PP2B)
**Calsequestrin**	Thermo Scientific	PA1-913	rabbit	polyclonal	1/1000	purified canine cardiac calsequestrin
**STIM1**	BD Laboratories	610954	mouse	monoclonal	1/500	amino acid 25–139 human
**Actin**	Santa Cruz	sc-1616	rabbit	polyclonal	1/500	C-terminus of Actin of human origin
**Desmin**	Sigma Aldrich	D1033	mouse	monoclonal	1/500	pig stomach was used as the immunogen
**Anti-Ca**_**v**_ **pan**	alomone	ACC-004	rabbit	polyclonal	1/200	C-terminus of rat Ca_v_1.2

### mRNA expression analysis using reverse transcription followed by PCR (RT-PCR)

For RT-PCR analysis, colonies were washed three times with ice cold PBS, snap-frozen in liquid nitrogen and stored at −70°C. Total RNA was isolated from myotubes using Qiagen RNeasy Mini Kit according to the instructions of the manufacturer (Qiagen, Valencia, CA USA). Assay mixture (20 μl) for reverse transcriptase reaction (Omniscript, Qiagen) contained 500 ng RNA, 0.25 μl RNase inhibitor, 0.25 μl oligo (dT), 2 μl dNTP (200 μM), 1 μl M-MLV RT in 1 × RT buffer. Amplifications of specific cDNA sequences were carried out using specific primer pairs that were designed by Primer Premier 5.0 software (Premier Biosoft, Palo Alto, CA, USA) based on human nucleotide sequences published in GenBank and purchased from Integrated DNA Technologies, Inc. (IDT; Coralville, IA, USA). The specificity of custom-designed primer pairs was confirmed *in silico* by using the Primer-BLAST service of NCBI (http://www.ncbi.nlm.nih.gov/tools/primer-blast/). Nucleotide sequences of forward and reverse primers and reaction conditions are shown in Table 2. PCR reactions were allowed to proceed in a final volume of 50 μl (containing 2 μl forward and reverse primers, 1 μl dNTP (200 μM), and 0.5 μl Promega GoTaq DNA polymerase (in 1 × reaction buffer) in a programmable thermocycler (Thermal Cycler C1000, Bio-Rad) with the following settings: 2 min at 95°C for initial denaturation followed by repeated cycles of denaturation at 94°C for 1 min, primer annealing for 60 s at an optimized temperature for each primer pair (see Table 2) and extension at 72°C for 1 min 30 s. After the final cycle, further extension was allowed to proceed for another 10 min at 72°C. PCR products were analyzed using 1.5% agarose gel at 100 V constant voltage.

**Table 2:** Nucleotide sequences, amplification sites, GenBank accession numbers, annealing temperatures and amplicon sizes for each primer pair are shown.

*Gene*	*Primer*	*Nucleotide sequence (5’→3’)*	*GenBank ID*	*Annealing temperature*	*Amplicon size (bp)*
*Skeletal muscle differentiaion*					
**Myostatin**	sense	ACTGGAATCCGATCTCTGAAACTT (665–688)	**NM_010834**	58.0°C	233
	antisense	GACCTCTTGGGTGTGTCTGTCAC (897–875)			
**MCIP1.4**	sense	AAGGAACCTCCAGCTTGGGCT (25–45)	**NM_019466.3**	60.0°C	160
	antisense	CCCTGGTCTCACTTTCGCTG (184–165)			
*Control Housekeeping gene*					
**GAPDH**	sense	AAGGTCGGAGTCAACGGATTTGG (99–121)	**NM_001289726.1**	56.0°C	322
	antisense	AATGAGCCCCAGCCTTCTCCAT (420–399)			

### Single cell fluorescent Ca^2+^-measurements

Measurements were performed using the calcium-dependent fluorescent dye Fura-2 as described previously [18]. Briefly, differentiated C2C12 skeletal muscle cultures were transferred to 1 mL fresh DMEM containing 5 μL Fura-2-acetoxy-methylester (AM; 10 μM; Life Technologies, Carlsbad, CA, USA) and 3 μL neostigmin (0.3 nM; to inhibit extracellular choline-esterase activity; TEVA, Debrecen, Hungary) and incubated in a CO_2_ incubator at 37°C for 1 h. Fura-2-loaded cells were then placed on the stage of an inverted fluorescent microscope (Diaphot; Nikon, Kowasaki, Japan) and viewed using a 40× oil immersion objective. Calcium imaging was performed in normal Tyrode’s solution (NTY; containing in mM, 137 NaCl, 5.4KCl, 0.5 MgCl_2_, 1.8 CaCl_2_, 11.8 HEPES; 1 gL^–1^ glucose; pH 7.4). KCl was applied in 120 mM final concentration in NTY by replacing an equal amount of NaCl. The sarcoplasmic/endoplasmic reticulum Ca^2+^-ATPase (SERCA) inhibitor cyclopiazonic acid (CPA) (10 μM) was diluted in Ca^2+^-free Tyrode’s solution (stock: 10 mM in DMSO). Cells were continuously washed with NTY using a background perfusion system excitation wavelength was altered between 340 and 380 nm (F_340_ and F_380_) by a microcomputer-controlled dual-wavelength monochromator equipment (DeltaScan; Photon Technologies International, New Brunswick, NJ, USA). Emission was detected at 510 nm at 10 Hz acquisition rate using a photomultiplier. Background fluorescence was subtracted on-line from F_340_ and F_380_ signals by the data acquisition software.

Intracellular [Ca^2+^] was calculated from the ratio of measured fluorescence intensities (R = F_340_/F_380_) as described by Grynkiewicz and colleagues [19]. The measuring bath was constantly perfused with NTY at a rate of 2 mLmin^–1^ (EconoPump; Bio-Rad Laboratories, CA, USA). Test solutions were directly applied to the cells through a perfusion capillary tube (Perfusion Pencil; AutoMate Scientific, San Francisco, CA, USA) with an internal diameter of 250 μm at a rate of 1.5 μLs^–1^, using a local perfusion system (Valve Bank 8 version 2.0, AutoMate Scientific). All measurements were performed at room temperature.

### Calculation of [Ca^2+^]_i_ and the activity of Ca^2+^-pump

[Ca^2+^]_i_ was calculated from the ratio of fluorescence intensities (*R* = *F*_340_/*F*_380_) using an *in vivo* calibration (*R*_min_ = 0.2045, *R*_max_ = 8.315, Kd × *β* = 1183 nM). To determine the activity of the Ca^2+^ pump and the Ca^2+^ flux entering the myoplasmic space, the Ca^2+^ binding to intracellular binding sites and the removal of Ca^2+^ from the intracellular space was modeled as presented in earlier reports [20,21]. In brief, Ca^2+^ binding to intracellular sites— to the SERCA pump, to troponin C, to parvalbumin, and to the dye —and the removal were considered. The computer routine determined, as a single best fit parameter, the maximal transport rate of the Ca^2+^ pump (PV_max_) from the declining phase of the Ca^2+^ transient following the stimulation. All other parameters in the model were held constant at values taken from the literature. The amount of calcium released from the SR was calculated as the sum of all calcium bound to myoplasmic binding sites and that transported by the SERCA pump [22].

### Examination of cell proliferation and differentiation

Cultures of C2C12 cells were photographed daily from the 1^st^ to the 5^th^ day after seeding using a Canon EOS-300D (Canon Corp., Japan) digital single lens reflex camera mounted on a phase contrast microscope. Culturing was performed in parallel in the presence of 200 nM cyclosporineA (CSA), solution was changed every 2^nd^ day. Photos of five fields of view per culture were taken every day. Myogenic nuclei were marked manually and morphometric analysis was done on these images. Cell proliferation was characterized by the increase in the number of myogenic nuclei normalized to the value obtained after 24 hours of culturing. To detect the quantitative parameters of differentiated myotubes 15–15 random fields of view were examined from 3 independent culturing (40x oil immersion objective, confocal laser scanning microscope). Cultures on the 5^th^ day of differentiation were fixed, and the number of DAPI-stained nuclei were counted manually, the diameter was measured by Image Browser.

### Calcineurin activity assay

For *in vitro* CaN activity assays, cells were washed in physiological NaCl solution and were harvested. After centrifugation, cell pellets were suspended in 100 μL of homogenization RIPA (Radio Immuno Precipitation Assay)-buffer (150 mM sodium chloride, 1.0% NP_4_0, 0.5% sodium deoxycholate; 50 mM Tris, pH 8.0) containing protease inhibitors (Aprotinin (10 ug/mL), 5 mM Benzamidine, Leupeptin (10 μg/mL), Trypsine inhibitor (10 μg/mL), 1 mM PMSF, 5 mM EDTA, 1 mM EGTA, 8 mM Na-Fluoride, 1 mM Na-orthovanadate). Samples were stored at—70°C. Suspensions were sonicated using pulsing bursts for 30 s at 40 A (Cole-Parmer, Illinois, USA). After centrifugation at 10,000×*g* for 10 min at 4°C, supernatants with equal protein concentrations were used for enzyme activity measurements. Activity of calcineurin was assayed by using RII phosphopeptide substrate, and the release of free PO_4_^3-^ was detected by a classic malachite green assay (Abcam, Cambridge, UK). Six separate wells were used from every single experimental group. Measurements were performed according to the instructions of the manufacturer in two independent experiments.

### Statistical analysis

All data are representative of at least three independent experiments. Averages are expressed as mean ± SEM (standard error of the mean; *n*, number of cells measured). Statistical analysis was performed by using Student’s *t*-test. Threshold for statistically significant differences as compared to respective control cultures was set at *p*<0.05.

## Results

### Cell culturing and transfection

**Fig 1:**
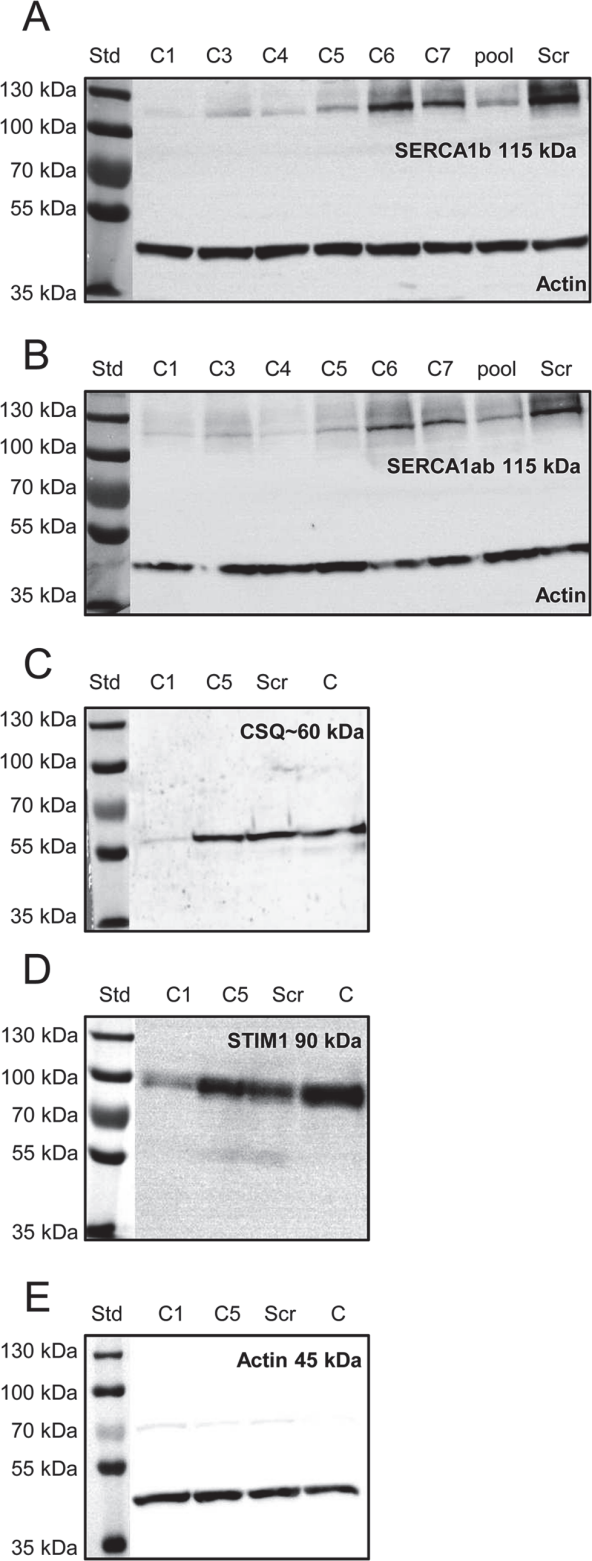
Effect of SERCA1b shRNA on the expression pattern of proteins involved in Ca^2+^-homeostasis. Examination of decreased SERCA1b expression in C2C12 myotubes. (**A-B**) Protein expression of SERCA was detected by Western-blot analysis to prove the efficiency of SERCA1b-specific shRNA in multinucleated myotubes. Different stably transfected clones, pool of the clones and scrambled shRNA transfected cells were compared. The 115 kDa isoforms were detected either by SERCA1b specific antibody corresponding to the terminal octamer of the protein or by an antibody recognizing both SERCA1a and b isoforms. Actin was used as a control. (**C**) Western blot analysis to detect the SR calcium-binding protein (calsequestrin) expression in selected cloneC1, C5, scrambled shRNA transfected, and parental cells. (**D**) Western-blot analysis showing the protein level of the key molecule of SOCE (STIM1). Total protein samples were used (30 μg in each lane) to examine the protein expression level. For preparing protein samples cultures were harvested on the 5^th^ day of differentiation in each cases. Representative data of 3 independent experiments.

The neonatal isoform SERCA1b is the major Ca^2+^-pump in myotubes and in muscle fibers from young animals [4,23]. The effect of SERCA1b silencing was thus examined in a mouse skeletal muscle cell line. SERCA1b protein synthesis has been interfered with using a specific shRNA sequence cloned into pLKO.1-puro-CMV-tGFP expression vector. Decreased protein expression was confirmed in the selected clones using a SERCA1b specific antibody at the myotube stage. As demonstrated in Fig 1A four of the six clones (C1, 3, 4, and 5) displayed marked suppression in SERCA1b expression. To investigate the possible compensatory overexpression of SERCA1a,– the adult sarcoplasmic reticulum ATPase isoform –, another antibody recognizing a common epitope of SERCA1a and SERCA1b was applied (Fig 1B). According to our observations the SERCA1 positivity was similar to SERCA1b expression pattern. Quantitative analysis of the Western-blots confirmed a very pronounced decrease in SERCA1b expression in certain identified clones compared to that of scrambled shRNA transfected cells (S1B Fig). CloneC1 and another clone (C5)– in which SERCA1b was downregulated to a lesser extent —were selected for further experiments (optical density values were 6.6±0.8% in cloneC1 and 14.7±1.9% in cloneC5 expressed as relative to that of scrambled shRNA transfected cells). Scrambled shRNA transfected cells were used as a control. All the experiments were performed on multinucleated, terminally differentiated myotubes on the 5^th^ day of differentiation.

### Reduced SERCA1b expression alters the expression of proteins involved either in the Ca^2+^-homeostasis or differentiation of skeletal muscle

**Fig 2:**
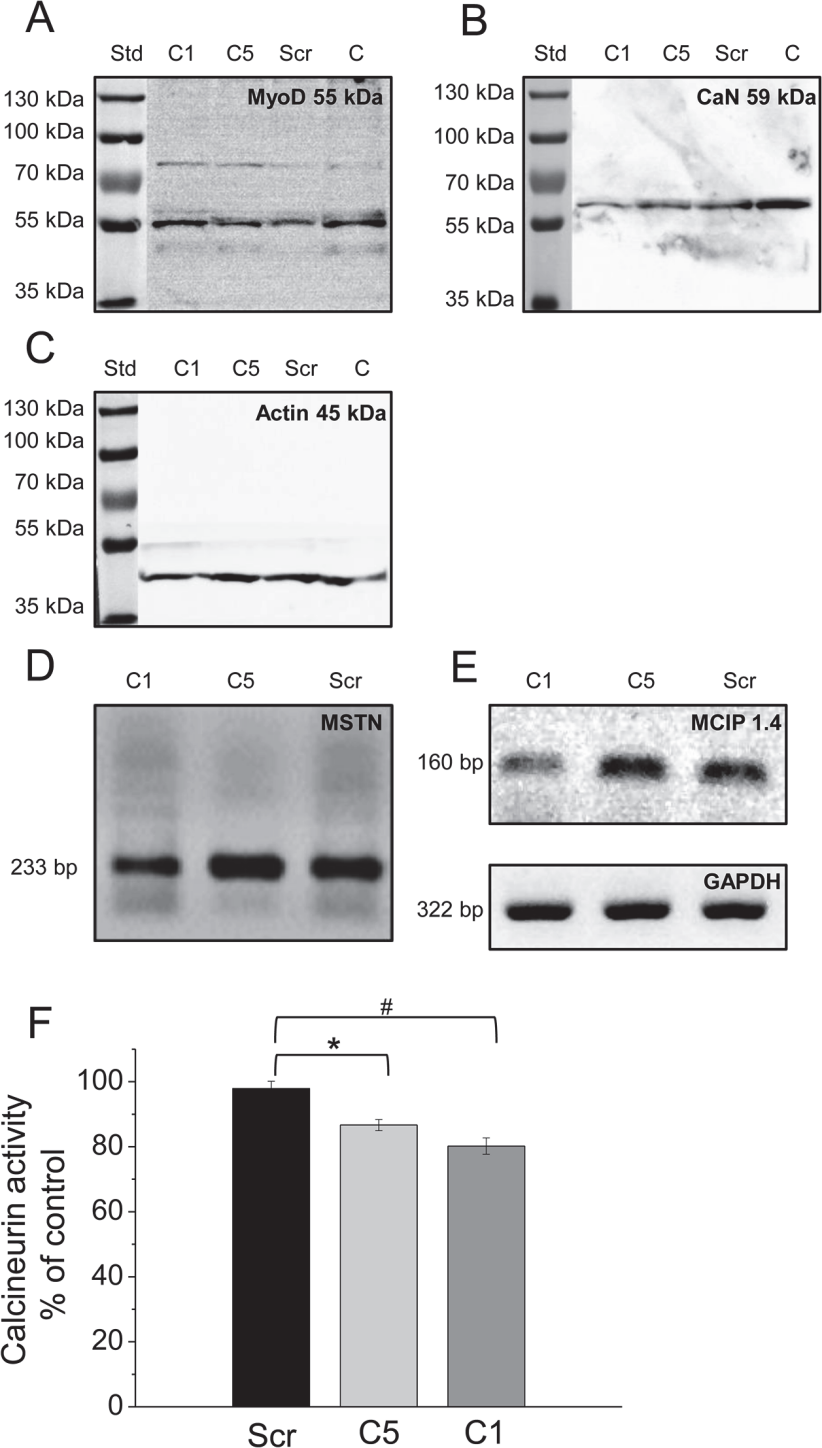
SERCA1b silencing modifies the proteins responsible for differentiation. Effect of decreased SERCA1b protein level on the expression pattern of key proteins involved in skeletal muscle differentiation. (**A-C**) Western-blot analysis to detect the main differentiation marker proteins (MyoD, and Calcineurin) on the 5^th^ day of differentiation. Total protein samples were used (30 μg in each lane) to examine the protein expression level. Representative data each of 3 independent experiments. Actin was used as a control. (**D-E**) mRNA expression pattern of myostatin and MCIP 1.4 was assessed by RT-PCR reaction using specific primers and detected at the expected size. GAPDH was used as a control. (**F**) Activity of calcineurin was assayed by using RII phosphopeptide substrate, and the release of free PO_4_^3-^ was detected by the malachite green assay. Six separate wells were used from every experimental group. Measurements were carried out in two independent experiments. Asterisks (*) mark significant (*P*<0.05) differences between cells.

Next the expression level of calsequestrin (CSQ)– the main Ca^2+^-binding protein inside the sarcoplasmic reticulum of skeletal muscle –and the stromal interacting molecule1 (STIM1)– the calcium sensor of SOCE in the SR —were studied by Western-blot analysis. The scrambled shRNA transfection did not modify the expression level of CSQ while, in the cloneC5 myotubes the expression showed a moderate decrease. Furthermore, in cloneC1 cells only a very weak band could be detected (Fig 1C). Similarly, STIM1 expression was slightly reduced in scrambled shRNA transfected and cloneC5 cells as compared to the parental cells, while the STIM1 expression was hardly detectable in cloneC1 (Fig 1D). Expression pattern of the main regulatory proteins that play an essential role in skeletal muscle differentiation were examined by Western-blotting and RT-PCR analysis. MyoD, and CaN were clearly detectable at protein level. The expression of the myogenic transcription factor (MyoD) was found to be unaffected. On the other hand, the calcium dependent phosphatase calcineurin showed a remarkable decrease in cloneC1 and even in cloneC5, as compared to control cell types (Fig 2A and 2B). Quantitative analysis of the results after normalizing to actin also confirmed the significant alterations in CSQ, STIM1, and CaN expression detected in cloneC1 as compared to control cells (S1C–S1F Fig). On the other hand, DHPR expression was similar in transfected and parental C2C12 cells (S2F Fig).

Using specific SERCA2a antibody to detect the isoform corresponding to the Ca^2+^pump in slow skeletal and cardiac SR, a band was detected with similar intensity at ~115 kDa in all C2C12 types. In mouse heart the SERCA2a positivity showed the adequate expression. (S2D and S2E Fig).

Using specific primer pairs, an mRNA transcript analysis of myostatin, – a negative regulator of skeletal muscle differentiation —and the modulatory calcineurin interacting protein, MCIP1.4 was performed. Amplimers of expected sizes were identified for all the available mRNAs, and myostatin showed a significantly decreased mRNA expression in cloneC1 as compared to scrambled shRNA transfected cells, thus the myostatin transcript level correlated with the SERCA1b silencing. In parallel MCIP1.4 was proved to be statistically modified in cloneC1 (Fig 2D and 2E). The optical density values of specific signals were normalized to GAPDH expression (S2A and S2B Fig; for raw data see Supporting Information—S1 Raw data).

Having confirmed the decreased expression of calcineurin, next the functionality of the protein was investigated. The application of a CaN activity assay revealed the significantly reduced activity of calcineurin in line with its decreased expression (Fig 2F).

### Effect of reduced SERCA1b expression, on Ca^2+^-homeostasis

**Fig 3:**
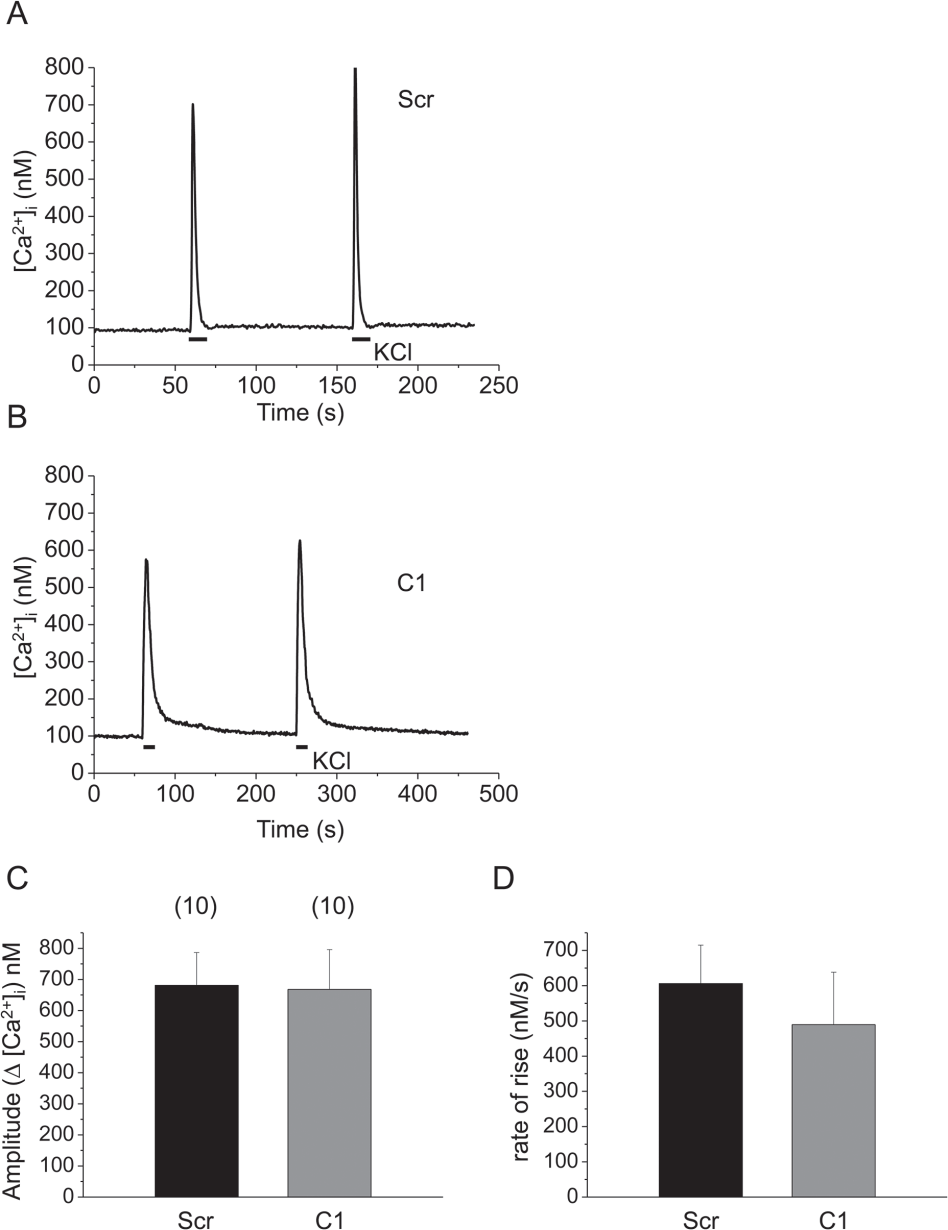
Functional effects of SERCA1b-specific shRNA transfection on depolarization-evoked calcium transients in C2C12 myotubes. Representative records of calcium transients evoked by repetitive application of 120 mM KCl in scrambled (**A**) and in SERCA1b-specific shRNA transfected (**B**) C2C12 myotubes (cloneC1). Calcium transients were detected in 1.8 mM Ca^2+^-containing Tyrode’s solution. (**C-D**) Pooled data of amplitudes and rates-of-rise of transients (calculated as maximal d[Ca^2+^]_i_/dt) evoked by 120 mM KCl. Numbers in parentheses indicate the number of cells measured. Data represent mean ± standard error of the mean (SEM).

To examine the possible alteration of E-C coupling and the depolarization induced Ca^2+^-release from the SR following SERCA1b gene silencing, repeated Ca^2+^-transients were evoked by the applications of 120 mM KCl in scrambled shRNA transfected and cloneC1 myotubes (Fig 3A and 3B). Neither the amplitude of the transients (681±105, n = 10 *vs*. 668±128 nM, n = 10 in control and in cloneC1 cells, respectively) nor their rate of rise (606±108 μM/s *vs*. 489±149 μM/s, respectively) were significantly altered (*p>0*.*5*) in cloneC1 myotubes (Fig 3C and 3D).

**Fig 4:**
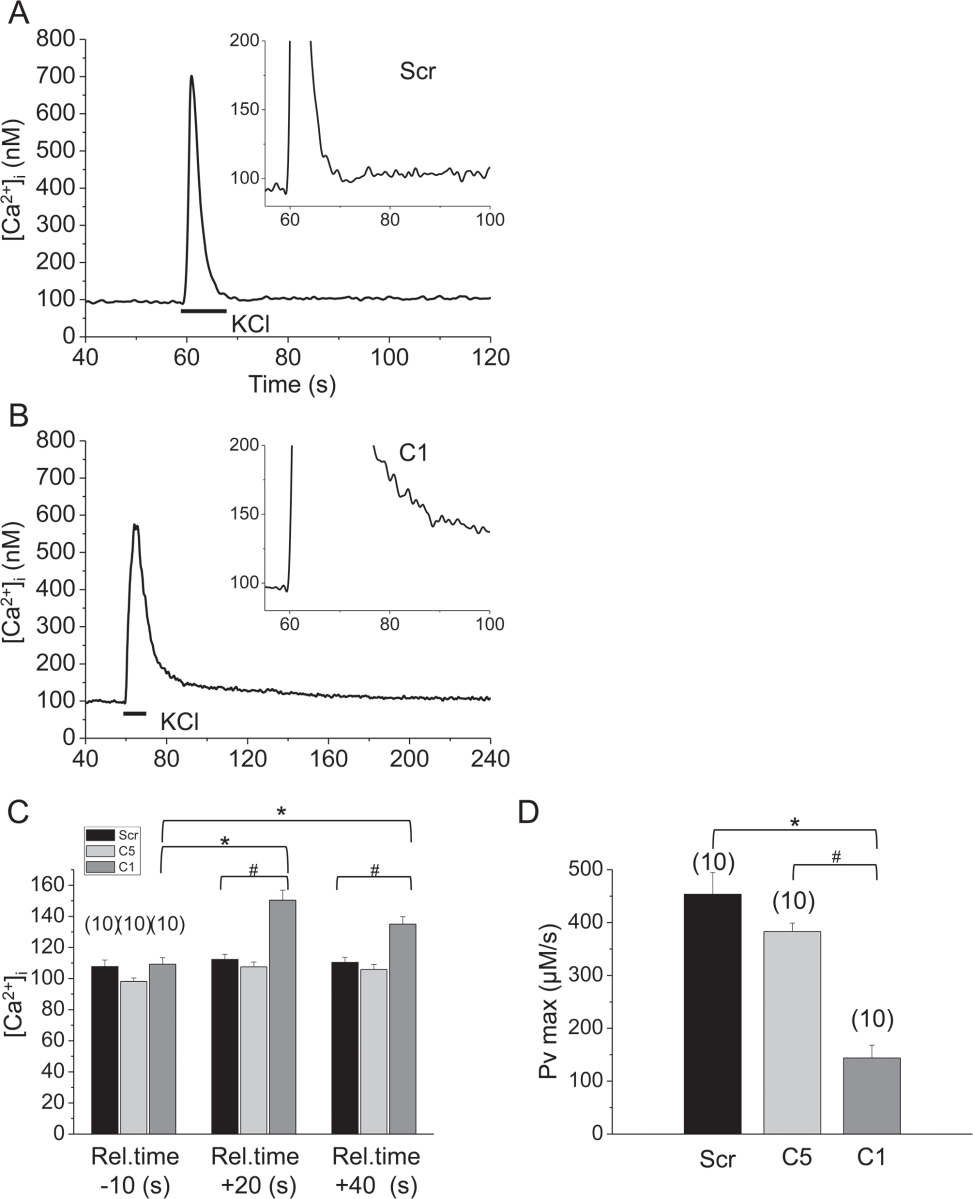
Detection of altered activity of the SERCA pump due to the decreased SERCA1b expression. Representative records showing the difference in maximal transport rate of the Ca^2+^ pump (PV_max_) from the declining phase of the Ca^2+^-transient following the KCl-induced depolarization (**A**) in scrambled and (**B**) in SERCA1b-specific shRNA transfected C2C12 myotubes (cloneC1). (**C**) Mean values of [Ca^2+^]_i_ before and after the transients, demonstrating that following the transients [Ca^2+^]_i_ declined slower and returned to a significantly higher level in cloneC1 compared to scrambled shRNA transfected cells. (**D**) Pooled data of SERCA pump activity in selected cloneC1, C5, and scrambled shRNA transfected cells. Numbers in parentheses indicate the number of cells measured. Asterisks (*) and Hashmarks (#) mark significant (*P*<0.01) differences between cells bathed in standard Tyrode’s solution.

To analyze the functional effects of decreased SERCA1b expression, the return of [Ca^2+^]_i_ to its resting value following the KCl-evoked transients and the maximal transport rate of the Ca^2+^ pump (PV_max_) were compared in scrambled shRNA transfected and cloneC1 and C5 myotubes. There was no difference in the resting [Ca^2+^]_i_ before the transients (108±4 nM in scrambled shRNA transfected and 109±4 nM in cloneC1 myotubes). However, following the KCl-evoked transients [Ca^2+^]_i_ declined slower and returned to a significantly higher level in the clone C1 myotubes (Fig 4A and 4B; note the different time scales and the insets).

A significantly higher [Ca^2+^]_i_ could be measured 20 and 40 seconds after the beginning of KCl application (112±3 nM, and 110±3 in control, while 150±7 nM and 135±5 in cloneC1 cells, respectively) indicating a decreased Ca^2+^-uptake capability of the SERCA pumps (Fig 4C). This was further quantified by extracting PV_max_(maximal transport rate of the pump; see Materials and methods), which was clearly decreased (p<0.01) in cloneC1 myotubes (144±24 μM/s) and in cloneC5 myotubes (382±16 μM/s) as compared to scrambled shRNA transfected cells (454±41 μM/s) in line with the decreased expression of SERCA (Fig 4D).

**Fig 5:**
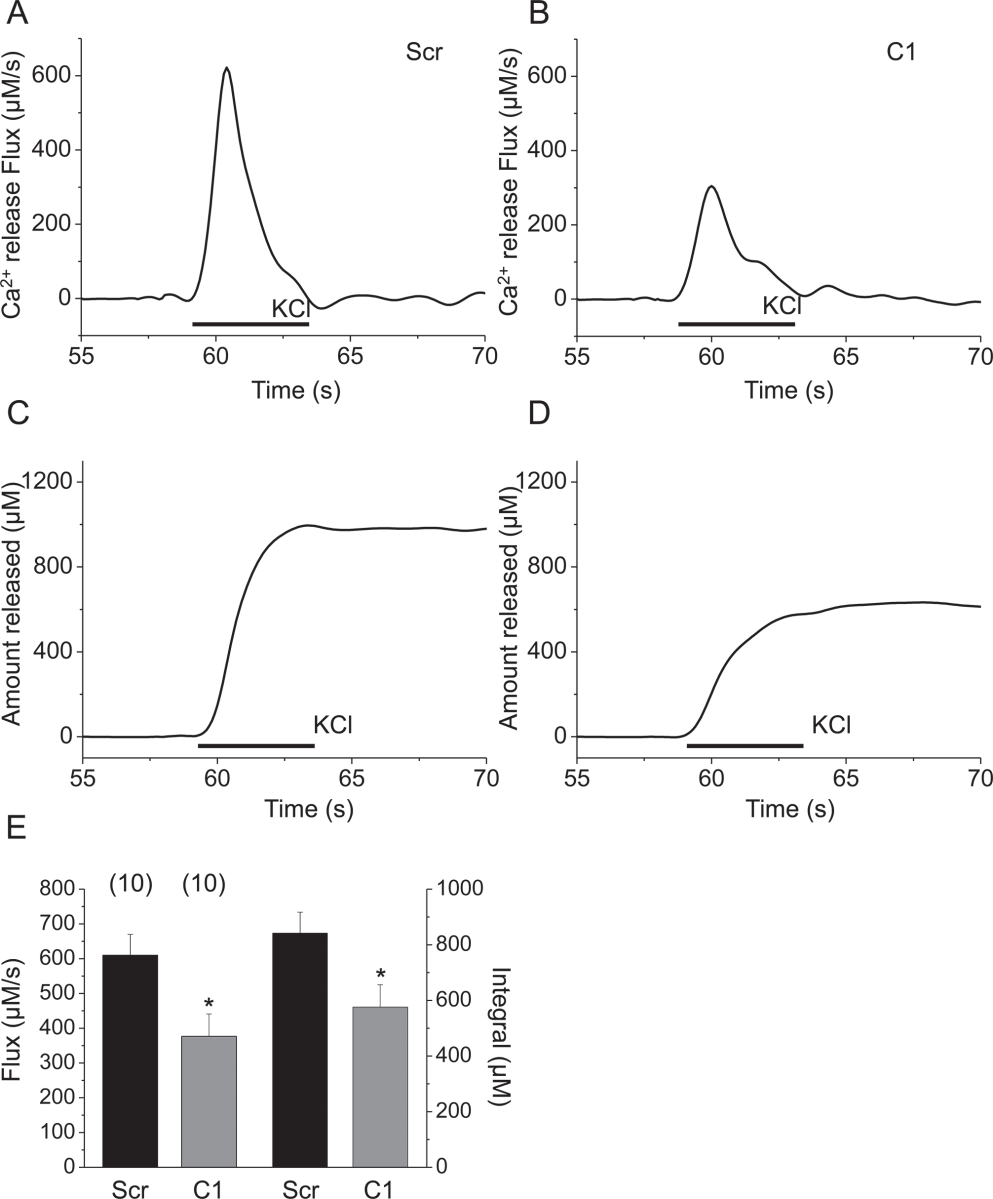
Effect of SERCA1b downregulation on releasable calcium measured on depolarization-evoked calcium transients. Representative records of calcium release flux (the amount of calcium entering the cytosol in one second) of (**A**) scrambled shRNA transfected and (**B**) cloneC1 myotubes. Representative records of the amount of calcium released from SR of (**C**) scrambled shRNA transfected and (**D**) cloneC1 myotubes. (**E**) Pooled data of calcium flux and released calcium in scrambled shRNA transfected and cloneC1 cells. Numbers in parentheses indicate the number of cells measured. Asterisks (*) mark significant (*P*<0.05) differences.

**Fig 6:**
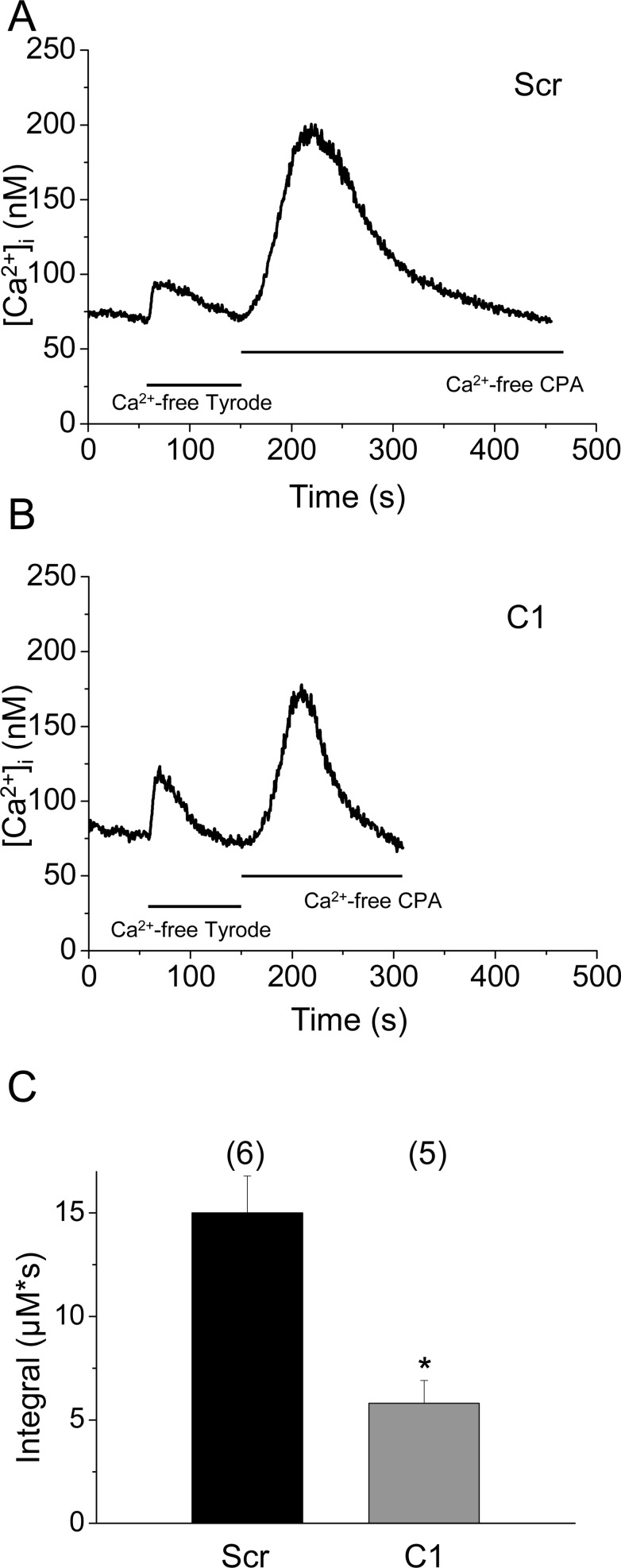
Comparison of releasable calcium. Internal Ca^2+^ stores of cells were depleted by the application of 10 μM CPA in Ca^2+^-free Tyrode’s, which caused an increase in [Ca^2+^]_i_.both (A) in scrambled shRNA transfected and (B) cloneC1 cells. (C) Mean values of the integral of CPA induced calcium transients. Numbers in parentheses indicate the number of cells measured. Asterisks (*) mark significant (*P*<0.05) differences.

It should be noted that despite of the significantly reduced removal rate of calcium from the myoplasmic space in cloneC1 cells (see values of PV_max_ above) the amplitude of the calcium transients in control and shRNA transfected cells were essentially identical (Fig 3C). In addition a slight reduction in the maximal rate-of-rise of KCl-evoked calcium transients was also observed (Fig 3D). These findings can only be reconciled if the amount of calcium released from the SR was reduced in cloneC1 cells. To test this hypothesis the Ca^2+^ release flux and the amount of calcium released were compared in scrambled shRNA transfected and cloneC1 C2C12 myotubes (Fig 5A and 5B). The calcium flux (610±60 *vs*. 377±64 μM/s, respectively) were suppressed significantly (*p<0*.*01*) as a result of SERCA1b silencing. Furthermore, the calculated integral for the releasable calcium (Fig 5C and 5D) was also proved to be significantly lower in cloneC1 myotubes as compared to scrambled shRNA transfected cells (843±75 μM *vs*. 576±80 μM; *p<0*.*02*). These observations suggest that the Ca^2+^-content of the SR and, consequently, the rate of Ca^2+^-release into the cytosol was decreased when the SERCA1b expression was interfered with (Fig 5E). To confirm this claim, further experiments were carried out. When the SERCA blocker CPA (10 μM) was administered to cells in Ca^2+^ free Tyrode’s, a pronounced elevation in resting Ca^2+^ levels was observed, caused by Ca^2+^ release from the SR *via* RyRs and concurrent inhibition of Ca^2+^ re-uptake (Fig 6A and 6B). From these data, the releasable Ca^2+^ content of internal Ca^2+^ stores could be calculated, which was significantly lower in C1 cells compared to scrambled shRNA transfected myotubes (5.8±1.1 μM*s and 15.7±1.8 μM*s; p<0.001) (Fig 6C) (for raw data see Supporting Information—S2 Raw data).

### SERCA1b silencing modifies the growth of differentiating skeletal muscle

**Fig 7:**
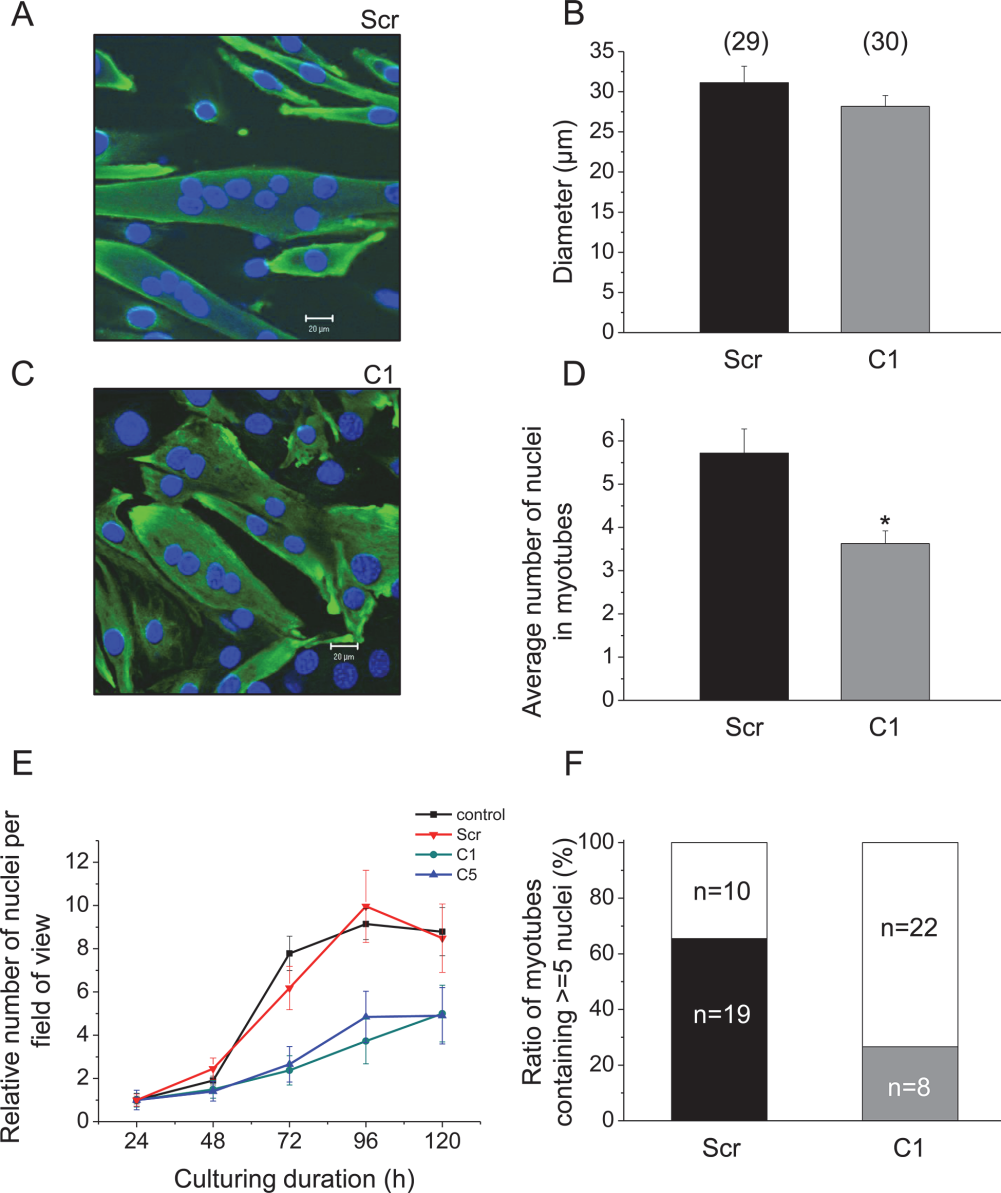
Effect of decreased SERCA1b expression on proliferation and differentiation of C2C12 cells. Immunocytochemical staining of terminally differentiated C2C12 myotubes demonstrating the morphological changes (**C**) in cloneC1 compared to (**A**) scrambled shRNA transfected cells. Muscle specific desmin was detected and visualized with FITC-conjugated secondary antibody. Nuclei were stained with DAPI. Images were recorded from1 μm thick optical slices. Original magnification was 40×. (**E**) Proliferation rate was calculated from the increase in the number of myogenic nuclei after normalising to the value obtained after 24 hours of culturing. Data represent mean ± standard error of the mean (SEM). (**B,D,** and **F**) Quantitative parameters of differentiated multinucleated myotubes.CloneC1 and scrambled shRNA transfected cells were compared. Numbers in parentheses indicate the number of identified myotubes on 3 different coverslips. Asterisks (*) mark significant (*P*<0.01) differences.

Morphological alterations of the cells in culture with decreased SERCA1b expression were also observed during the proliferation and differentiation period, and were compared to those transfected with the scrambled shRNA. Multinucleated myotubes were treated with anti-desmin primary antibody and visualized with FITC conjugated secondary antibody (Fig 7A and 7C). Quantitative parameters of myotubes cultured under the same conditions were analyzed on the 5^th^ day after exchanging the proliferating medium to differentiating medium which corresponded to the terminal stage of differentiation. The diameter of terminally differentiated cloneC1 myotubes did not differ significantly (p>0.2) from that of the control (Fig 7B). In contrast, the average number of nuclei was significantly decreased (5.7±0.5 in scrambled shRNA transfected cells and 3.6±0.2 in cloneC1 cells, p<0.01, Fig 7D). In line with this result, myotubes containing 5 or more nuclei could be detected in a lower ratio than it was observable in control cultures (Fig 7F). These results confirmed the observations obtained visually, that is, decreased SERCA1b expressing C2C12 cells form less differentiated myotubes. To investigate the proliferation rate, 10,000 cells were plated on the 0^th^ day of culturing. On the 4^th^ day of culturing the rate of proliferation— the number of myogenic nuclei normalized to that counted on the 1^st^ day —was significantly lower (p<0.05) in the cloneC1 cultures, as compared to parental cells (3.7±1.1 and 9.1±0.7, respectively; Fig 7E), indicating that these cells were dividing at a slower rate (for raw data see Supporting Information—S3 and S4 Raw data).

Since the expression and activity of CaN was found to be reduced in cloneC1 myotubes, the effect of CSA was investigated on the rate of proliferation of C2C12 cells (S2C Fig). CSA significantly inhibited the proliferation of parental cells as indicated by the relative number of myogenic nuclei on the 4^th^ day (9.1±0.7 *vs*.5.6±0.6; in control and CSA treated cells, respectively; p<0.05). On the other hand, CSA did not further decrease the rate of proliferation in cloneC1 cultures (3.6±0.5 *vs*. 3.7±1.1; in CSA treated and non-treated cloneC1 cells, respectively; p>0.4) indicating that the reduced proliferation seen in these cells was likely to be the consequence of their suppressed CaN activity (for raw data see Supporting Information—S5 Raw data).

## Discussion

The neonatal sarcoplasmic/endoplasmic reticulum Ca^2+^-ATPase (SERCA1b) was successfully silenced in several clones of the mouse myogenic cells stably transfected with shRNA expressing vector. Because of the close homology between mouse and rat the sequence of shRNA was the same as the one which induced growth stimulation during the regeneration of rat soleus muscle after being transfected by a plasmid in a previous study [15]. Since the transfection of regenerating rat muscle was effective only in a few fibers the silencing could not be controlled in the whole muscle extract only by SERCA1b immunostaining in the fibers and this method was difficult to evaluate quantitatively [15]. Apart from this, the efficiency of silencing was demonstrated at the mRNA level by co-expressing SERCA1b and the shRNA in COS-1 cells [15]. Therefore the present work provides the first clear evidence that the sequence spliced together from exon 21 and 23 of the SERCA1 transcript can be targeted for efficient silencing of the neonatal SR Ca^2+^-pump protein in myogenic cells. The immunoblot signal of total SERCA1 declined in parallel with that of SERCA1b in C2C12 cells, showing that the expression of the other muscle specific SERCA1 isoform, SERCA1a is not upregulated in compensation for the SERCA1b silencing. This is in accordance with an earlier work showing that SERCA1b is the only considerable SERCA1 isoform in C2C12 cell line [4]. It is also important to note that the expression of the adult and neonatal splice isoforms are under strict translational control in developing or regenerating rodent muscle; practically no SERCA1a protein is found in myoblasts and myotubes and, *vice versa*, no SERCA1b protein has been found in denervated or stretched adult muscles in spite of its increased mRNA level [4].

The other muscle specific SR calcium pump in C2C12 cells, SERCA2a is expressed from a different gene (atp2a2), however, its expression level was again not elevated in response to SERCA1b silencing. The later phenomenon again complies with the observation that not SERCA2a but SERCA1b protein level increases significantly at the time of myotube development in both regenerating slow (*m*. *soleus*) and fast type (*m*. *EDL*) muscles [4,24,25]. The fusion of myoblasts into myotubes is paralleled with ER/SR maturation (reviewed by [8]). SERCA1b is found at a low level in myoblasts but its expression is much higher in C2C12 myotubes suggesting its importance in myotubes and SR development [4]. Myoblasts, as most other cell types express SERCA2b which has a lower ATPase activity and a higher affinity for Ca^2+^ than the slow muscle specific isoform SERCA2a spliced from the transcript of the same gene [26,27,28], reviewed by [28]. Because of its higher Ca^2+^ transporting capacity and Ca^2+^-dependence, SERCA2a is a more relevant pump to reduce the elevated intracellular Ca^2+^ level than SERCA2b. However, SERCA2a has about half of the Ca^2+^ transport capacity and ATPase activity of SERCA1a as demonstrated in COS-1 cells [29]. Interestingly, no such differences have been found between SERCA1a and SERCA1b in these cells [11]. This might partially explain why SERCA1b and not SERCA2a protein content is elevated at the time of myotube development in both slow and fast regenerating muscle [24,25].

**Fig 8:**
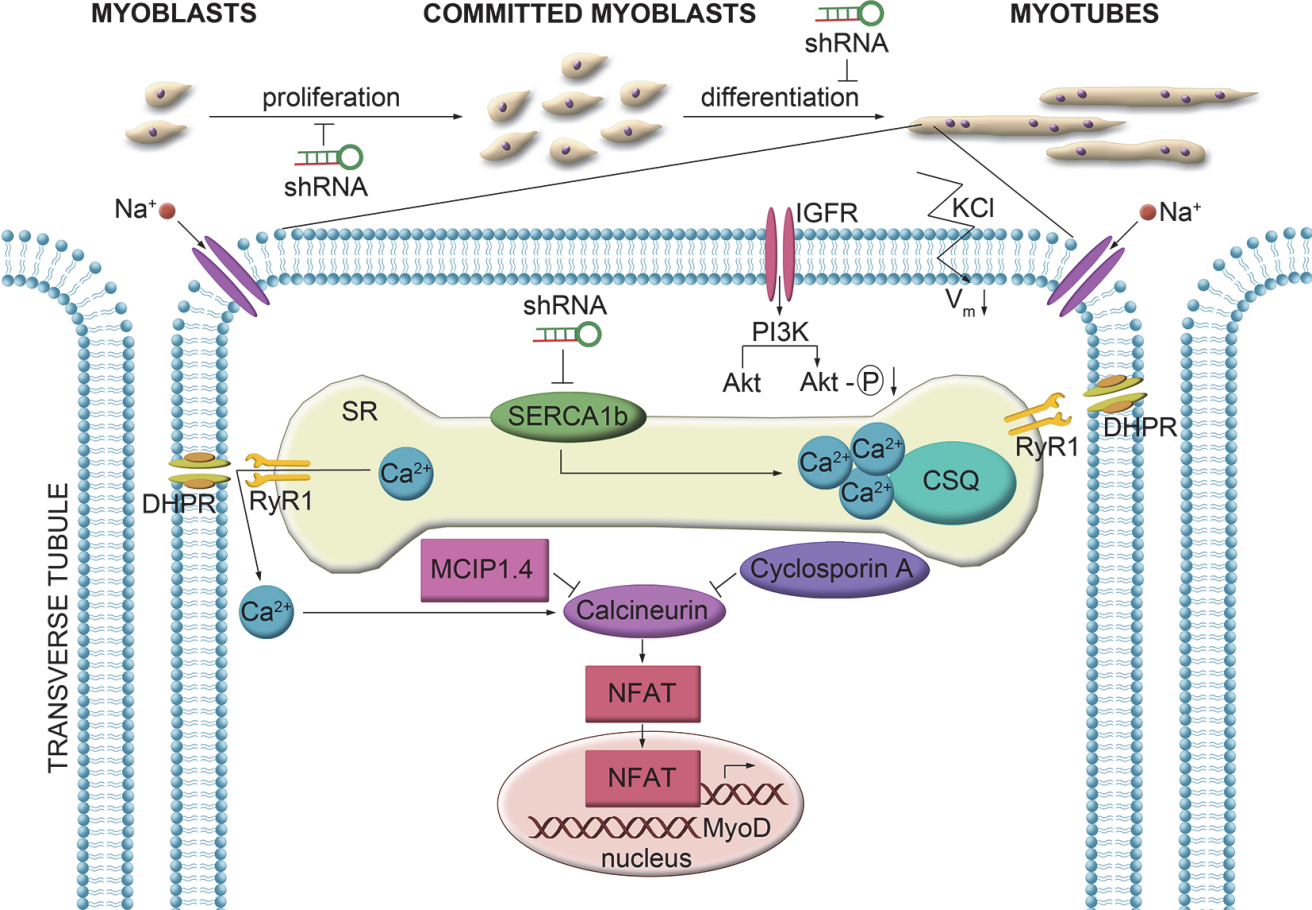
Summarized model showing the role of SERCA1b, in the regulation of calcium homeostasis. Application of shRNA interfering with the SERCA1b expression resulted in a lower Ca^2+^-content of the SR and suppressed Ca^2+^-release into the myoplasm. In turn the activity of calcineurin- a calcium-dependent protein phosphatase which induces the translocation of the transcription factors of the NFAT family into the nucleus-was attenuated. The reduced proliferation and differentiation capability of myoblasts could therefore be attributed to a decreased activation of muscle specific genes under the control of NFAT.

When the ER/SR is filled up with calcium from the extracellular source by SOCE (reviewed by [8]), the elevation of myoplasmic calcium level is slower but more sustained than in case of excitation-contraction coupling and it is able to activate calcineurin, a serine threonine phosphatase [30]. CaN in turn dephosphorylates transcription factors, as NFAT isoforms and inducing their translocation into the nucleus where they activate muscle specific differentiation genes [30,31]. Accordingly, the level and activity of the calcium-dependent calcineurin was also decreased even in a less (C5) not just in the most silenced clone (C1). However, the MCIP1.4 mRNA level, produced from a CaN-target gene and used as an activity marker of CaN [32,33,34] declined only in the most effectively SERCA1b silenced (cloneC1) myotubes indicating an indirect and less prompt response than CaN. CSQ, the main Ca^2+^-binding protein in the SR lumen also declined only in the most silenced clone (C1) implicating a lower need of binding SR Ca^2+^ (Fig 8).

STIM1, a key element of SOCE is also crucial for muscle development since STIM1 defective myotubes are lower in number, have low levels of SR Ca^2+^, and fatigue rapidly [7,35]. In addition, sarcolipin— an endogenous peptide inhibitor of SERCA1 —has a markedly increased level in STIM1 defective myotubes, sarcolipin, therefore, also contributes to the reduction in SOCE and thus delays myotube development [35]. As we pointed out earlier, the SERCA1b isoform is probably responsible for most of the SERCA1 (in myotubes [4]). Recently Lee et al. reported that STIM1 binds to SERCA1a and stimulates its activity in rabbit skeletal muscle and in primary mouse myotubes [17]. Such binding can only happen to a peptide sequence that must also be present in SERCA1b since SERCA1b contains the entire sequence of SERCA1a except the C-terminal glycine [3]. Generating an antibody is not feasible against a C-terminal glycine since it can be present in many other proteins besides SERCA1a. However, Lee et al. [17] claim to identify SERCA1a by an antibody that was otherwise specified by the source company as a SERCA1 antibody (recognizing both SERCA1a and SERCA1b) Lee et al. [17]therefore might well have demonstrated the STIM1 effect on SERCA1b, because they worked with primary mouse myotubes that probably have SERCA1b as the main SERCA1 isoform. Anyway the present work is in agreement with the importance of SERCA1b in SOCE because the level of the calcium sensor STIM1 was decreased in SERCA1b silenced myotubes. It is a general observation that elements of SOCE are regulated by a positive feedback during myotube development, *i*.*e*., decreased or increased levels of NFAT isoforms (NFATc1-c4) are paralleled by decreased or increased levels of STIM1 and Orai1 [7,35,36]. It is worth noting that the STIM1-knockdown primary mouse myotubes also had decreased SERCA1 activity [17]. It is, therefore, feasible to suggest that the lower level of STIM1 and SERCA1b generates lower rate of Ca^2+^ movement across the myoplasm which in turn stimulates less calcineurin and results in reduced downstream gene activity reported by the MCIP1.4 mRNA level. Similarly, the silenced SERCA1b could fill the SR to a lower Ca^2+^ level requiring, therefore, a lesser amount of CSQ for storage. On the other hand, the silencing did not modify the myogenic transcription factor MyoD level suggesting that SERCA1b itself is not directly coupled to MyoD in the myogenic differentiation. This is in agreement with the observation that the expression of MyoD is started earlier (in myoblasts, [37]) than that of SERCA1b (in myotubes, [4]) during the differentiation of C2C12 cells. However, the reduced transcript level of myostatin in cloneC1 cells suggest a positive interaction with SERCA1b expression. Although myostatin has not been reported to be directly related to SOCE, our recent findings with hypermuscular mice [38] suggest that this compact phenotype may be correlated with altered SOCE. We can thus assume that the decreased level of this factor occurs as part of the mechanism of myotube growth attenuation.

To examine the effects of SERCA1b silencing on excitation-contraction coupling, repeated Ca^2+^ transients were evoked. Neither the amplitude nor the rate of raise of the transients in cloneC1 myotubes was different from those measured in the scrambled control cells indicating that the function of RyR and the voltage-gated channels was not likely affected. Exposure to high K^+^ concentration triggered similar Ca^2+^ elevations in the sarcoplasmic space of both SERCA1b silenced and non-silenced myotubes. This indicates that both cell types have a developed SR filled up to some extent with calcium [39]. Noteworthy in this context that the calcium was evidently reuptaken into the SR even in the SERCA1b silenced myotubes indicating that other SERCA pumps (most probably SERCA2a) also take part in this process. However, as it is concluded from the relative time and the extracted maximal uptake capacity, the SERCA1b silenced myotubes restored the sarcoplasmic calcium back to the normal level much slower. This indicates that SERCA2a, the only other intact high capacity Ca^2+^pump, is not capable of normally restoring the sarcoplasmic calcium level by itself without the neonatal Ca^2+^ pump; therefore the SERCA1b is also essential in maintaining normal reuptake speed in these myotubes.

On the other hand, the relatively normal Ca^2+^ elevation in the sarcoplasm and the slower Ca^2+^ removal from the sarcoplasm in silenced myotubes could not be reconciled without assuming a decreased amount of released Ca^2+^ into the sarcoplasmic space and without a lower Ca^2+^ influx, which was indeed found. These parameters together enlighten a lower releasable SR Ca^2+^ content in silenced than in control myotubes. This suggests that SERCA1b significantly contributes to the refilling of the SR with normal amounts of calcium. Similar results have been obtained in SERCA1 knock-out mice (where neither SERCA1a nor SERCA1b is expressed), namely, the Ca^2+^ reuptake was slower. [12] As a consequence [12] the contractile force was lower in the diaphragm and in the hind limb muscles, while SERCA2a expression was also not changed in compensation to the ablation of SERCA1 [12]. It is noteworthy in this context that 1–5 hours after birth, when SERCA1 knock-out mice usually die, SERCA1b is still the dominantly expressed isoform in the corresponding muscles of wild type mice [4]. The changes in the Ca^2+^ homeostasis of SERCA1b silenced C2C12 cells is also in agreement with the suggestion that the lack of the neonatal Ca^2+^ pump might be responsible for the effects seen in SERCA1 KO mice. Cell proliferation is sensitively connected to SOCE and SERCA (reviewed by [40,41]). In accordance, the more (cloneC1) and even the less (cloneC5) efficiently silenced cells showed inhibited muscle cell proliferation and fewer myogenic nuclei in their cultures as compared to that of control cells. The necessity of SERCA1b for cell proliferation seems to be connected to calcineurin regulated signaling pathway since CSA, a calcineurin inhibitor, decreased cell proliferation in the parental non-transfected cells, but surprisingly no further decrease of proliferation was observed in the most silenced clone (cloneC1). It should be noted here, that the specificity of CSA to calcineurin can be cell type dependent as it can enhance neuronal cell survivor ([42]) but it can induce nephrotoxicity in a calcineurin independent way ([43]). CSA have been used as a potent calcineurin inhibitor in osteoblast cell line ([44]) or in chondrogenic cell cultures ([45]). Furthermore, it has been discussed that the inhibition of calcineurin with CSA can modulate the phenotype of muscles ([46]). Moreover, calcineurin can modulate the proliferation of smooth muscle cells ([47]) and the inhibition with CSA reduce the proliferation ratio of chondrogenic cells ([48]). This is in line with the observations that CSA abolished slow muscle differentiation and regeneration and, to a lesser extent, myoblast proliferation [49,50]. Our finding that CSA did not inhibit the proliferation in SERCA1b silenced myogenic cell lines suggests that either the CSA-dependent component of calcineurin activity was mostly based on SERCA1b activity or raise the possibility of a calcineurin independent CSA effect.

Difference between myotube nuclear numbers of the most silenced cell line and of the one transfected with scrambled oligos was also significant. A similar result was obtained in an earlier work [51] when thapsigargin, a potent SERCA inhibitor was added to the medium of C2C12 cells and this treatment reduced the rate of myogenic differentiation. Myotube formation is based on the density of myoblasts in a certain field. First the primary myotubes are formed with 4–6 nuclei then the secondary myotubes develop further accumulating nuclei from fusing myoblasts preferably at their end [52]. SERCA1b silenced myotubes (cloneC1) show a strong incline of having less than five nuclei. This suggested a relatively undisturbed primary myotube formation and an inhibited secondary myotube development. This is in agreement with our previous results that BC3H1 myogenic cells that are reluctant to form long (secondary) myotubes with more than four nuclei are not expressing SERCA1b, only SERCA1a [4]. The result also demonstrates that SERCA2a alone is not able to compensate completely for the declined (cloneC5) or abolished (cloneC1) SERCA1b in myoblast proliferation, only for the SERCA1b decline (cloneC5) in myotube development. SERCA2a the only other high capacity SR calcium pump in myogenic cells [53] may also show an increasing expression in C2C12 differentiation [54]. Therefore the neonatal SR calcium pump seems to be more important for myoblast proliferation and secondary myotube formation than for primary myotube development.

In summary, SERCA1b is required for myoblast proliferation and secondary myotube formation in murine C2C12 myogenic cells by having an important role in Ca^2+^ homeostasis (Fig 7). During this process its expression is coupled to those of STIM1, CSQ, and calcineurin suggesting a role in SOCE, too.

## Supporting Information

S1 Fig Localization of SERCA1b specific shRNA (A) corresponding to the mRNA of *mus musculus* SR ATPase. Quantitative analysis of Western-blot experiments. (**B**) Quantified expression of SERCA1b in specific shRNA transfected C2C12 clones. (**C-F**) Quantified expression of proteins involved in Ca^2+^-homeostasis and differentiation of skeletal muscle. CloneC1, C5, and scrambled shRNA transfected control cells were compared to parental cells in each cases. Asterisks (*) indicate significant (*p* < 0.05) differences. Representative data of 3 independent experiments.
(DOCX)

S2 Fig mRNA expression pattern of MSTN and MCIP1.4 were detected by RT-PCR. (**A, B**) Pooled data represent integrated optical densities of signals determined by ImageJ after normalizing to GAPDH as a control (**C**) Proliferation rate as calculated from the increase in the number of myogenic nuclei normalised to the value obtained after 24^h^ culturing duration, when the cells were treated with 200 nM CSA. Data represent mean ± standard error of the mean (SEM). Data for parental and cloneC1 without CSA treatment are also presented at 125^th^ hour of culturing. (**D**) Expression pattern of SERCA2a in cloneC1, C5, scrambled shRNA transfected, and parental C2C12 cells. Actin was used as loading, while lysate from adult mouse heart was used as expression control. (**E**) Quantitative analysis of SERCA2a expression, transfected cells were compared to parental cells. (**F**) Expression pattern of voltage dependent calcium channel (Ca_v_ pan α1 subunit) in cloneC1, C5, scrambled shRNA transfected, and parental C2C12 cells. Reference figure of Alomone Labs is also attached. Representative data of 3 independent experiments.
(DOCX)

S1 Raw Data Raw data of densitometric analysis of Western-blot experiments. Quantification of bands referring to the detected proteins was performed with ImageJ.
(XLSX)

S2 Raw Data Raw data of single cell fluorescent Ca^2+^-measurements. Ca^2+^ transients were evoked by repeated KCl-depolarization. To analyze the functional effects of decreased SERCA1b expression, the return of [Ca^2+^]_i_ to its resting value following the KCl-evoked transients and the maximal transport rate of the Ca^2+^ pump (PV_max_) were compared. To examine the Ca2+ content of the SR Ca2+ store the cells were treated with CPA.
(XLSX)

S3 Raw Data Raw data of calculation of cell proliferation. Cell proliferation was characterized by the increase in the number of myogenic nuclei normalized to the value obtained after 24 hours of culturing.
(XLSX)

S4 Raw Data Raw data of examination of morphology of myotubes. Cultures on the 5^th^ day of differentiation were fixed, and the number of DAPI-stained nuclei were counted manually, the diameter was measured by Image Browser.
(XLSX)

S5 Raw Data Activity of calcineurin was assayed by using RII phosphopeptide substrate, and the release of free PO_4_^3-^ was detected by a classic malachite green assay (Abcam, Cambridge, UK). Six separate wells were used from every single experimental group.
(XLSX)
