# Supplementary figures and images for: The Effect of SERCA1b Silencing on the Differentiation and Calcium Homeostasis of C2C12 Skeletal Muscle Cells
Source: PLoS One. 2015 Apr 20;10(4):e0123583. doi: 10.1371/journal.pone.0123583 (PMC4404259; doi:10.1371/journal.pone.0123583)

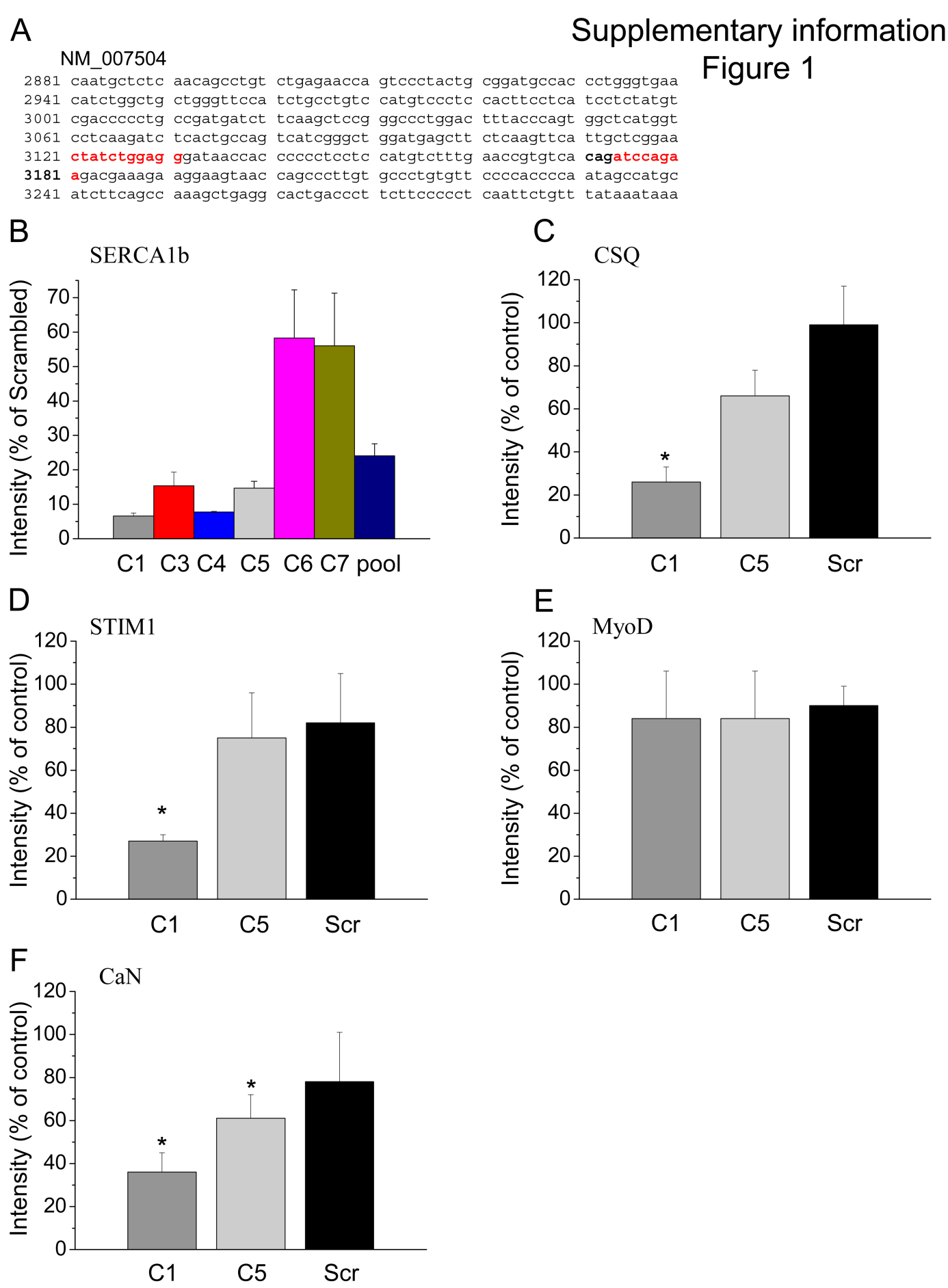

Supplement: S1 Fig — Quantitative analysis of Western-blot experiments. (B) Quantified expression of SERCA1b in specific shRNA transfected C2C12 clones. (C-F) Quantified expression of proteins involved in Ca2+-homeostasis and differentiation of skeletal muscle. CloneC1, C5, and scrambled shRNA transfected control cells were compared to parental cells in each cases. Asterisks (*) indicate significant (p < 0.05) differences. Representative data of 3 independent experiments. (DOCX) [file pone.0123583.s001.docx]

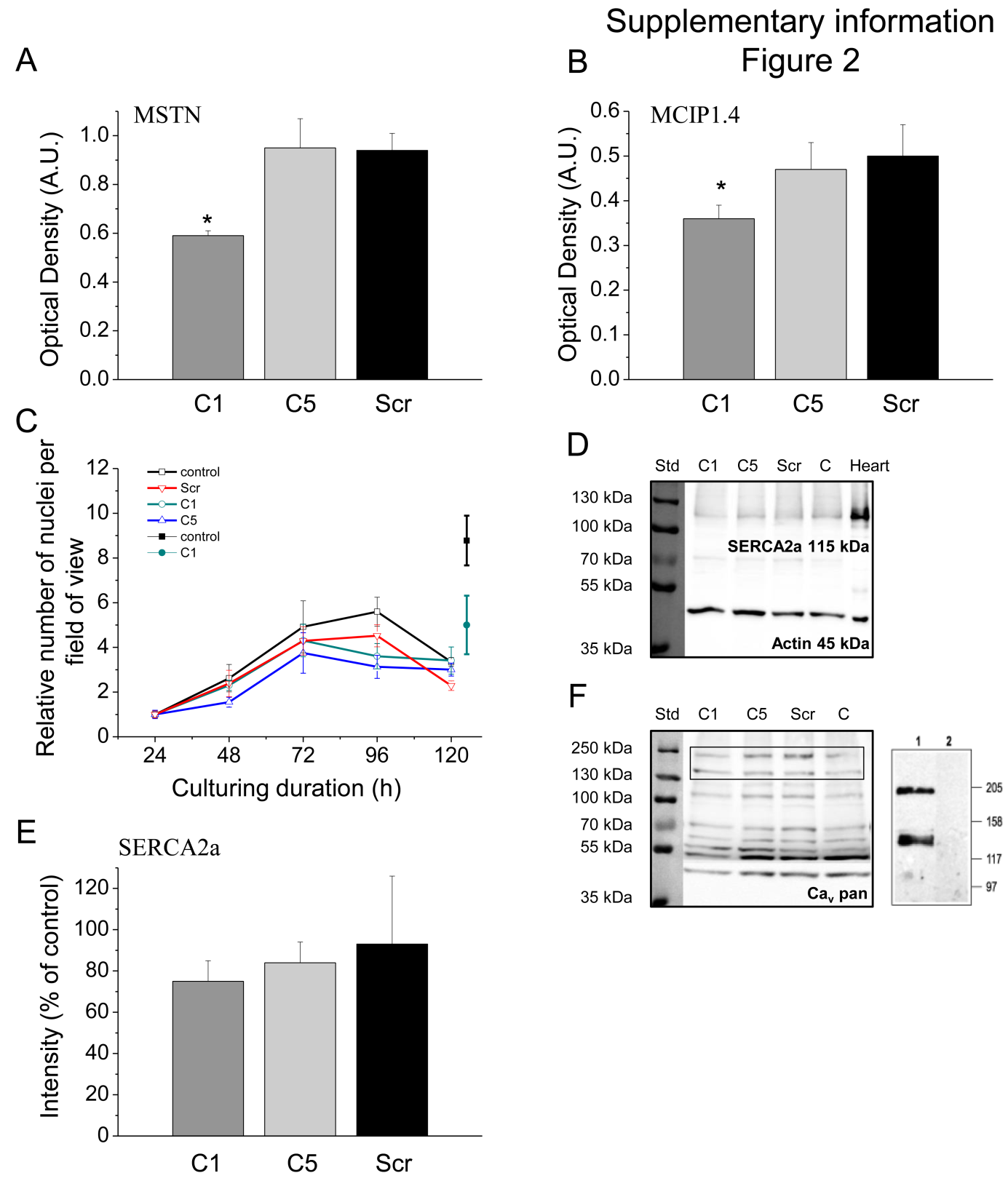

Supplement: S2 Fig — (A, B) Pooled data represent integrated optical densities of signals determined by ImageJ after normalizing to GAPDH as a control (C) Proliferation rate as calculated from the increase in the number of myogenic nuclei normalised to the value obtained after 24h culturing duration, when the cells were treated with 200 nM CSA. Data represent mean ± standard error of the mean (SEM). Data for parental and cloneC1 without CSA treatment are also presented at 125th hour of culturing. (D) Expression pattern of SERCA2a in cloneC1, C5, scrambled shRNA transfected, and parental C2C12 cells. Actin was used as loading, while lysate from adult mouse heart was used as expression control. (E) Quantitative analysis of SERCA2a expression, transfected cells were compared to parental cells. (F) Expression pattern of voltage dependent calcium channel (Cav pan α1 subunit) in cloneC1, C5, scrambled shRNA transfected, and parental C2C12 cells. Reference figure of Alomone Labs is also attached. Representative data of 3 independent experiments. (DOCX) [file pone.0123583.s002.docx]
